# MIF gene rs755622 polymorphism positively associated with acute coronary syndrome in Chinese Han population: case–control study

**DOI:** 10.1038/s41598-019-56949-z

**Published:** 2020-01-10

**Authors:** Guo-Li Du, Jun-Yi Luo, Duolao Wang, Yan-Hong Li, Bin-Bin Fang, Xiao-Mei Li, Xiao-Ming Gao, Yi-Ning Yang

**Affiliations:** 1 State Key Laboratory of Pathogenesis, Prevention and Treatment of High Incidence Diseases in Central Asia, Department of Cardiology, First Affiliated Hospital of Xinjiang Medical University, Urumqi, China; 2 Department of Endocrinology, First Affiliated Hospital of Xinjiang Medical University, Urumqi, China; 3 Xinjiang Key Laboratory of Medical Animal Model Research, Urumqi, China; 4 Xinjiang Key Laboratory of Cardiovascular Disease Research, Urumqi, China; 5 Department of Clinical Sciences, Liverpool School of Tropical Medicine, Liverpool, L3 5QA United Kingdom; 6 Department of Clinical Laboratory, First Affiliated Hospital of Xinjiang Medical University, Urumqi, China

**Keywords:** Acute coronary syndromes, Cardiovascular genetics

## Abstract

Macrophage migration inhibitory factor (MIF) has been recognized as a major player in the pathogenesis of atherosclerosis. This study determined the association between polymorphisms of MIF gene and acute coronary syndrome (ACS). The polymorphism of MIF gene (rs755622, rs1007888 and rs2096525) was analyzed in 1153 healthy controls and 699 ACS cases in Chinese Han population. Plasma MIF level was also measured in part of ACS patients (139/19.9%) and healthy controls (129/11.2%) randomly. Most participants including healthy controls and ACS patients carried rs755622 GG (63.1% vs. 56.7%) and CG genotypes (33.1% vs. 38.9%) and G allele of rs755622 (79.6% vs. 76.1%, respectively), while CC genotype (3.8% vs. 4.4%) and C allele (20.4% vs. 23.9%) carriers were the lowest. Multivariate logistic regression analysis showed that carriers with rs755622 C allele had a higher risk of ACS compared to other genotypes (AOR = 1.278, 95% CI: 1.042–1.567). In addition, CC genotype carriers had the highest plasma levels of MIF than other genotype carriers. The MIF level in ACS patients with CC genotype was significantly higher than ACS patients carrying GG genotype and healthy controls carrying 3 different genotypes of MIF gene rs755622. Our findings indicate that MIF gene rs755622 variant C allele is associated with increased risk of ACS. Identification of this MIF gene polymorphism may help for predicting the risk of ACS.

## Introduction

Coronary artery disease (CAD) is the leading cause of mortality worldwide, accounting for about 30% of deaths globally in 2012 and approximately 70% of deaths in developing countries^1^. Acute coronary syndrome (ACS) is an urgent condition of CAD due to a rupture of the atherosclerotic plaque in coronary arteries. The complexity of CAD pathogenesis poses significant challenges to decision making for effective interventions. Use of multi-marker algorithms including biological markers and genetic markers will improve prediction of CAD risk in clinic^2^.

Inflammation plays critical roles in CAD and participates pivotally in almost every stage of atherosclerosis including initiation, progression and destabilization of plaque and myocardial infarction^3^. MIF is produced by different cell types such as immune cells (monocytes, macrophages and lymphocytes) and endocrine, endothelial and epithelial cells^4^. It has been proved that MIF plays an essential role in a variety of acute and chronic inflammatory disorders^5,6^ as well as in cancer^7,8^. Previous studies have also revealed a critical role of MIF in atherosclerosis^9,10^, therefore, MIF is very likely associated with the risk of ACS.

The human MIF gene locates at chromosome region 22q11.2 comprising approximately 800 nucleotides and containing 3 exons and 2 introns^11^. At least five single nucleotide polymorphisms (SNPs) in the human MIF gene locating at -173G/C (rs755622), +254 (rs2096525), +656 (rs2070766), 3.8 kb 3′ of the translation termination codon (rs1007888) and -794 CATT_5-8_ microsatellite have been mainly reported. Loci rs2096525 and rs2070766 locate at introns, rs755622 and -794 CATT_5-8_ locate at the promoter region of MIF and rs1007888 located in the 3′ flanking region of the MIF gene. Of them, -173G/C (rs755622) and -794 CATT_5-8_ have been reported to be associated with CAD^12–14^.

Xinjiang, the northwest part of China, is a region with multiple ethnic populations. People living in Xinjiang have some special life styles and habits which are different from other regions of China, for example more meat, dairy products and alcohol consumption. Our previous epidemiological investigation revealed the prevalence of hypertension (33.4%^15^ vs. 27.9%^16^), dyslipidemia (53.3%^17^ vs. 40.4%^16^), type-2 diabetes (9.26%^18^ vs. 5.5%^19^) and obesity [(1082/5757, unpublished data) vs. 11.9%^16^] in Chinese Han population in Xinjiang were higher than other parts of China. It is expected that a higher prevalence of CAD in Xinjiang with an association of the higher prevalence of all major risk factors i.e. hypertension, dyslipidemia, diabetes and obesity. Moreover, a significant difference in prevalence of major cardiovascular risk factors and adverse risk profiles among different ethnic groups in Xinjiang has been reported^20^. We have previously demonstrated that there was a significant association between the polymorphism of MIF gene -173G/C (rs755622) and CAD and severity of coronary lesions in Chinese Kazakh population^13^. Considering potential genetic influence and MIF as a key factor in atherosclerotic plaque formation, in this study, we investigate, in Han Chinese population, whether the variants of MIF gene predispose to ACS and have function influence on circulating plasma MIF level in both healthy controls and ACS patients.

## Materials and Methods

### Ethic declaration

This study was approved by the Ethics Committee of the First Affiliated Hospital of Xinjiang Medical University (Urumqi, China) and conducted according to the standards of the Declaration of Helsinki and written informed consents were obtained from all of the participants.

### Study population

In this unmatched case-control study, we recruited adult patients with ACS patients from Chinese Han population who lived in Xinjiang from 2008 to 2013. The patients presenting with persistent chest pain more than 20 min, typical electrocardiographic changes including new pathologic Q waves and ST segment elevation more than 1 mm, and increased plasma levels of creatinine kinase-MB isoenzyme (CK-MB) (more than 2-fold higher than the upper reference limit) and/or troponin-T more than 0.1 μg/ml according to the guidelines^21^. All ACS patients included underwent coronary angiography to confirm the ACS diagnosis, exemplified with identifiable culprit-vessel, i.e. ≥50% luminal stenosis in at least one coronary artery or major branch segments. Patients were excluded if they had regional wall motion abnormalities, relevant valvular abnormalities in echocardiograms, acute inflammatory diseases, tumors, autoimmune disease and severe hepatic and renal dysfunction.

The control participants were recruited from the cardiovascular risk survey study and had no evidence of cardiovascular and other systemic disease as determined on history, physical examination, blood test and ECG recording. The design of this study has been previously reported^13,22^.

### Biochemical assays

Venous blood samples were drawn at the catheter laboratory before angiography from ACS patients and from healthy controls during medical examination. Blood biochemistry including total cholesterol (TC), triglyceride (TG), low-density lipoprotein-cholesterol (LDL-C) and high-density lipoprotein-cholesterol (HDL-C) in all participants were performed using the commercially available automated platform (Dimension AR/AVL Clinical Chemistry System, DADE Bchring, Newark, NJ), in the Central Laboratory of the First Affiliated Hospital of Xinjiang Medical University. We also randomly selected some ACS patients and healthy controls to test plasma MIF levels by using enzyme linked immunosorbent assay (ELISA). The randomization of participants selection was computer generated at the Key Laboratory of Cardiovascular Disease Research of Xinjiang Medical University (blocked randomization) and the participants’ allocations were kept in sequentially numbered and sealed envelopes. Plasma MIF levels were measured using Quantikine MIF ELISA kits (R&D Systems, USA) according to manufacturer’s specifications at Xinjiang Key Laboratory of Cardiovascular Disease Research.

### Polymorphisms selection

A total of three SNPs (rs755622, rs1007888, and rs2096525) of the MIF gene whose minor allele frequencies (MAF) are more than 0.1 were selected from the HapMap human SNP database (www.hapmap.org). The rs755622 locates in the promoter region, rs1007888 locates in the translation termination codon and rs2096525 locates in the first intron of MIF gene. We also found that the above three SNPs were the tagging SNP of the Chinese Han population (MAF ≥ 0.1 and with r^2^ ≥ 0.8 as a cut-off in linkage disequilibrium pattern analysis) in the HapMap.

### Genotyping of MIF gene

Genomic DNA was extracted from the peripheral leukocytes using standard phenol-chloroform method and stored at −80 °C for future analysis. We analyzed the polymorphism of MIF gene using the TaqMan® SNP genotyping assay (Applied Biosystems). The primers and probes used in the assay were chosen according to the information at the ABI website (http://myscience.appliedbiosystems.com). We utilized the Applied Biosystems7900HT Standard Real-Time PCR System to amplify genomic DNA. The results of each polymorphism of MIF gene were read by the Sequence Detection Systems (SDS) automation controller software v2.3 (ABI). The reaction system of PCR amplification was as follows: 3 μL of TaqMan Universal Master Mix, 0.12 μL probes and 1.88 ddH_2_O in a 6 μL final reaction volume containing 1 μL DNA (50 ng). Amplification cycling conditions were as follows: 95 °C for 5 min; 40 cycles of 95 °C for 15 s and 60 °C for 1 min. Samples with ambiguous genotypes that were not separated by discrete clusters were re-genotyped as we described previously^13^.

### Definition of Cardiovascular Risk Factors

The formula, body weight (kg)/height (m^2^) was used to calculate the body mass index (BMI). Overweight/obesity was classified as a BMI ≥ 24 kg/m^2^. Hypertension was defined as history of hypertension and/or repeated systemic blood pressure measurements exceeding 140/90 mmHg. Diabetes was defined as history or presence of diabetes and/or a fasting plasma glucose level ≥ 7.0 mmol/L (126 mg/dL) or a random glucose value ≥ 11.1 mmol/L (200 mg/dL) or a 2-hour plasma glucose ≥ 11.1 mmol/L during an oral glucose tolerance test (OGTT) plus signs or symptoms of diabetes. If there is no signs or symptoms of diabetes, the glucose level should be examined on another day. Concentrations of glucose ≥ 6.1 mmol/L, TC ≥ 5.20 mmol/L, TG ≥ 1.71 mmol/L, LDL-C ≥ 3.10 mmol/L, and HDLC < 1.04 mmol/L were defined as hyperglycemia, hypercholesterolemia, hypertriglyceridemia, hyper-LDL-C or hypo-HDL-C, respectively.

### Statistical analyses

Data analysis was performed using the SPSS version 21.0 (Chicago, IL, USA). We calculated the sample size according to the formula of unmatched case-control study^23^ and previous data^13^. Continuous variables were expressed as mean ± standard deviation (SD) and compared using the unpaired Student t test between ACS group and control group. Categorical variables were compared using the Chi-square test. We also used Chi-square test in Hardy-Weinberg equilibrium (HWE) to compare the frequencies of genotypes and alleles between ACS patients and control participants.

We used logistic regression models to analyze association between MIF gene polymorphisms and ACS in two steps. We first fitted a univariate logistic regression model to evaluate association between rs755622 together each of the variables listed in Table 1 and ACS. We then perofrmed a multivariate logistic regression model to assess the association between MIF gene polymorphisms and ACS controlling for possible confounding factors used in the univariate analysis. Crude odds ratios (COR) and adjusted odds ratios (AOR) were calculated along with 95% confidence intervals (CIs). Statistical significance was set at *P* < 0.05.

**Table 1:** Demographic and clinical characteristics of patients with acute coronary syndrome (ACS) patients and healthy control group.

Characteristics	Control (n = 1153)	ACS (n = 699)	*P*-Value
Age (years)	58.7 ± 11.1	59.1 ± 10.1	0.001
Male, n (%)	635 (55.1)	431 (61.7)	0.005
BMI (kg/m^2^)	25.7 ± 3.1^*^	26 ± 2.9	0.028
Overweight and obesity (BMI ≥ 24 kg/m^2^)	795 (69.0)^*^	531 (76.0)	0.001
Smoking, n (%)	834 (72.3)	439 (62.8)	<0.001
Hypertension, n (%)	719 (37.6)	348 (50.2)	<0.001
Diabetes, n (%)	953 (27.7)	479 (37.2)	<0.001
Glucose (mmol/L)	5.25 ± 1.5	5.61 ± 1.61	<0.001
Glucose ≥ 6.1 mmol/L, n (%)	171 (14.8)	174 (24.9)	<0.001
TG (mmol/L)	1.56 ± 0.59	1.58 ± 0.55	<0.001
TG ≥ 1.71 mmol/L, n (%)	398 (34.5)	252 (36.1)	0.503
TC (mmol/L)	4.4 ± 0.64	4.58 ± 0.75	<0.001
TC ≥ 5.2 mmol/L, n (%)	136 (11.8)	154 (22.0)	<0.001
HDL–C (mmol/L)	1.06 ± 0.22	1.03 ± 0.19	<0.001
HDL–C < 1.04 mmol/L, n (%)	610 (52.9)	313 (44.8)	0.001
LDL–C (mmol/L)	2.32 ± 0.53	2.52 ± 0.62	<0.001
LDL–C ≥ 3.1 mmol/L, n (%)	97 (8.4)	127 (18.2)	<0.001

Continuous variables are expressed as mean ± SD. Categorical variables are expressed as percentages. BMI, body mass index; TG, triglyceride; TC, total cholesterol; HDL–C, high–density lipoprotein–cholesterol; LDL–C, low density lipoprotein–cholesterol. *One participant was missing the measurement of height.

## Results

### Demographic and clinical characteristics of participants

A total of 699 ACS patients (mean age 59.1 ± 10.1 years and 61.7% men) and 1153 healthy controls (mean age 58.7 ± 11.1 years and 55.1% men) were recruited in the present study. Demographic and clinical characteristics of all participants are summarized in Table 1. ACS patients were older and had greater BMI and levels of glucose, LDL-C and TG and higher prevalence of hypertension, diabetes, hyperglycemia, hypercholesteremia and hyper-LDL-C but lower HDL-C level compared to healthy controls (all *P* < *0*.*05*).

### MIF gene polymorphism rs755622 positively associated with ACS

Three SNPs of MIF (rs755622, rs1007888, and rs2096525) were successfully genotyped in all ACS patients and healthy controls (Table 2). The MAF of rs755622, rs1007888 and rs2096525 were 0.239, 0.483 and 0.212, respectively. All the genotype frequencies were in Hardy-Weinberg equilibrium (HWE, *P* > *0*.*05*). There was no significant difference in distribution of genotypes or alleles rates in rs1007888 and rs2096525 between ACS and control groups (*P* > *0*.*05*). However, we found significant differences of genotypic and allelic distribution in rs755622. The frequency of the CC genotype in ACS patients was higher than that in control subjects (4.4% vs. 3.8%, *P* = *0*.*024*). Moreover, the frequency of the C allele was also higher in the ACS than that in control group (23.9% vs. 20.4%, *P* = *0*.*012*).

**Table 2:** Association analyses between genotypes and alleles of the MIF gene polymorphisms in patients with acute coronary syndrome (ACS) and in healthy control group.

Polymorphisms	Control	ACS	*P*-Value
	n = 1153	n = 699	
***rs755622***			
GG	727 (63.1%)	396 (56.7%)	0.024
CG	382 (33.1%)	272 (38.9%)	
CC	44 (3.8%)	31 (4.4%)	
G allele	1836 (79.6%)	1064 (76.1%)	0.012
C allele	470 (20.4%)	334 (23.9%)	
***rs1007888***			
AA	273 (23.7%)	150 (21.5%)	0.264
AG	574 (49.8%)	375 (53.6%)	
GG	139 (23.1%)	84 (26.3%)	
A allele	1120 (48.6%)	675 (48.3%)	0.866
G allele	1186 (51.4%)	723 (51.7%)	
***rs2096525***			
TT	694 (60.2%)	426 (60.9%)	0.736
CT	413 (35.8%)	250 (35.8%)	
CC	46 (4.0%)	23 (3.3%)	
T allele	1801 (78.1%)	1102 (78.8%)	0.603
C allele	505 (21.9%)	296 (21.2%)	

Univariate regression analysis showed that the C allele in rs755622 was the risk factor for ACS (CG and CC genotype vs. GG genotype, OR = 1.306, 95% CI, 1.078–1.581, *P* = *0*.*006*). Multivariate logistic regression analysis was further used to detect the association between rs755622 polymorphisms and susceptibility of ACS. After adjusting the confounding factors including age, gender, BMI, smoking, hypertension, diabetes, TG, TC, HDL–C and LDL–C, the C allele remained the independent risk factor for ACS (CG and CC genotype vs. GG genotype, AOR = 1.278, 95% CI, 1.042–1.567, *P* = *0*.*019*, Table 3). However, no difference was observed in the other two SNPs.

**Table 3:** Univariate and multivariate analysis of effects of MIF SNP rs755622 and characteristics of subjects on the risk of acute coronary syndrome.

Characteristics	N	COR (95% CI)	P	AOR (95% CI)	P
**rs755622**					
GG	1123	Ref.			
CG + CC	729	1.306 (1.078–1.581)	0.006	1.278 (1.042–1.567)	0.019
Age	1852	1.009 (0.995–1.013)	0.409		
**Gender**					
Female	786	Ref.			
Male	1066	1.312 (1.083–1.589)	0.005	1.086 (0.842–1.402)	0.524
**Smoking**					
No	579	Ref.			
Yes	1273	1.548 (1.267–1.892)	<0.001	1.498 (1.153–1.946)	0.002
**BMI** ≥ **24** **kg/m**^2^					
No	525	Ref.			
Yes	1326	1.419 (1.146–1.758)	0.001	1.257 (0.997–1.584)	0.053
**Hypertension**					
No	785	Ref.			
Yes	1067	1.671 (1.382–2.021)	<0.001	1.294 (1.054–1.591)	0.014
**Diabetes**					
No	420	Ref.			
Yes	1432	2.189 (1.755–2.729)	<0.001	3.692 (2.754–4.949)	<0.001
**Glucose** ≥ **6**.**1** **mmol/L**					
No	1507	Ref.			
Yes	354	1.903 (1.503–2.410)	<0.001	1.045 (0.790–1.381)	0.760
**TG** ≥ **1**.**71** **mmol/L**					
No	1202	Ref.			
Yes	650	1.069 (0.879–1.302)	0.503		
**TC** ≥ **5**.**2** **mmol/L**					
No	290	Ref.			
Yes	1562	2.113 (1.641–2.721)	<0.001	1.975 (1.504–2.594)	<0.001
**HDL-C** < **1**.**04** **mmol/L**					
No	923	Ref.			
Yes	929	0.722 (0.598–0.872)	0.001	0.832 (0.679–1.020)	0.077
**LDL-C** ≥ **3**.**1** **mmol/L**					
No	1761	Ref.			
Yes	89	3.462 (2.207–5.430)	<0.001	1.965 (1.443–2.675)	<0.001

BMI, body mass index; TG, total triglyceride; TC, total cholesterol; HDL–C, high–density lipoprotein–cholesterol; LDL–C, low density lipoprotein–cholesterol; COR, crude odds ratio; AOR, adjusted odds ratio.

### Plasma MIF levels significantly associated with MIF gene rs755622 polymorphisms

**Figure 1:**
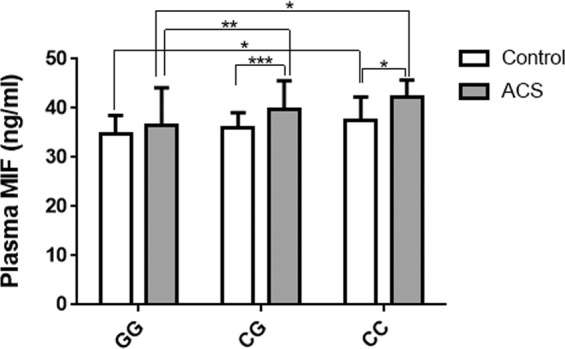
Influence of the MIF gene polymorphism rs755622 on plasm MIF levels in patients with acute coronary artery syndrome (ACS) and healthy controls. Comparison of plasm MIF levels among different genotypes of MIF gene rs755622 polymorphism. Values are expressed as mean ± SD, *P < *0*.*05*, **P < *0*.*01* and ***P < *0*.*001*.

The relationship between plasma MIF levels and MIF gene rs755622 polymorphisms (GG, CG and CC genotypes) were further evaluated. CC genotype carriers had the highest plasma levels of MIF than CG and GG genotype carriers. The MIF level in ACS patients with CC genotype was 6.5% and 15.6% higher than that in ACS patients carrying CG and GG genotypes, respectively. MIF levels in ACS patients carrying C allele (both in CG and CC phenotypes) were also significantly higher than healthy controls carrying the same phenotypes (Fig. 1, all *P* < *0*.*05*).

## Discussion

Many factors involve in the development and progression of the coronary artery atherosclerosis. Inflammation is one of the most important risk factors in the pathogenesis of ACS. Variations in multiple genes involved in inflammation, and previous studies showed that there were close relationships between PPARα, interleukin 18 (IL-18), IL-1β, SIRT2, and CD14 receptor and incidence of acute myocardial infarction^24–27^. In the present study, we found the polymorphism (rs755622) of MIF gene was significantly associated with the risk of ACS in Chinese Han population.

MIF was initially identified more than 6 decades ago as T-cell–derived cytokine with a function to inhibit random migration of macrophages *in vitro*^28^. Over the years, MIF has been generally believed to be a multifaceted cytokine and a critical mediator of the host immune response and inflammation. Previous studies show that MIF is a key modulator which promotes and modulates the magnitude of the acute inflammatory response, such as acute lung injury^29^, acute kidney injury^30^, acute adipose tissue inflammation^31^ and acute rheumatic fever^32^. MIF has been also proved to be associated with vulnerability of atherosclerotic plaque^9,33^. Several studies have documented that MIF regulates inflammatory cell recruitment to lesion areas through interaction with the chemokine receptors CXCR2 and CXCR4^34^. MIF is highly expressed in vulnerable plaques and it can enhance expression and activity of matrix metalloproteinases (MMPs), thereby contributing to destabilization of the atherosclerotic plaque^34,35^.

In this case–control study, we evaluated the associations between the MIF genetic variants (rs755622, rs1007888, and rs2096525) and ACS in Chinese Han population living in Xinjiang. We found that participants with rs755622 GC and CC genotypes were more susceptible to ACS than those with GG genotype allele after adjustments for potential confounding factors. A similar finding was also reported in a small case-control study conducted in other Chinese population^36^. Our previous study reported that Chinese Kazakh carrying MIF rs755622 CC genotype or C allele had an increased risk of CAD^13^. Some studies conducted in other races also found a close association between MIF gene polymorphism and CAD. In ONICA/KORA case-cohort study, carriers of the minor alleles rs755622C and rs2070766G had a higher risk for incident CAD in southern Germany^37^. In a western Mexican population, association between MIF -794 (CATT)_5-8_ polymorphism and susceptibility of ACS was detected^14^. This information indicates that MIF is a promising genetic marker for predicting the risk of CAD. However, some negative findings were also reported. A study conducted in Czech and Russian population failed to detect a significant difference in the distribution of MIF -173G/C genotypes, alleles or carriage rates between patients with myocardial infarction and control groups^38^. The lack of association between CAD class and MIF -794 (CATT)_5-8_ polymorphisms with soluble MIF levels in a small number of CAD subjects (n = 70) was reported^39^. Although the reason for these discrepant results is not defined, it may be due to the difference in the stage of CAD and phenotypic variance, therefore a large scare of clinical study is warranted to confirm the universal implication of MIF gene polymorphism in predicting the risk of CAD.

Further, in the present study, we also measured plasma MIF levels in part of ACS patients and healthy controls. We found that CC phenotype carriers had the highest MIF levels. MIF levels in ACS patients carrying C allele (both in CG and CC phenotypes) were significantly higher than healthy controls carrying the same phenotypes and also higher than ACS patients carrying GG phenotype. Similar findings were also reported in a small case-control study with a higher plasma MIF level in MIF-173C carriers than in MIF-173G carriers^36^. Another study including 138 ACS and 98 stable angina pectoris (SAP) patients documented that ACS patients had significantly higher plasma MIF levels compared with SAP patients^33^. In both ACS and SAP patients, plasma MIF levels increased in conjunction with the extent of complex lesions^33^. Our previous study in Chinese Kazakh population also revealed that CAD patients with rs755622 C allele (CC and CG genotype) have higher levels of Gensini score when compared to C allele noncarriers^13^. These findings further demonstrate the functional link between MIF gene polymorphism, especially -173C (rs755622) carrying and associated MIF production during occurrence of ACS and CAD and the severity of coronary lesion. Indeed, our previous study showed that plasma MIF levels in ST-segment elevated myocardial infarction (STEMI) correlated with cardiac magnetic resonance (CMR)-derived infarct size, ventricular volumes and ejection fraction, suggesting that plasma MIF levels are predictive of final infarct size and the extent of cardiac remodeling^10^.

Taken together, MIF gene polymorphism in general population may bear predictive value for the risk of CAD, while in ACS or CAD patients MIF gene polymorphism with CC genotype and C allele carrying in conjunction with plasma MIF levels may add values for predicting the severity of CAD and even prognosis.

### Study Limitations

There are several limitations in this study. *First*, we only studied the association between three SNP of MIF gene (rs755622, rs1007888 and rs2096525) and risk of ACS in Chinese Han population in Xinjiang and did not cover the other variations of the MIF gene such as -794 (CATT)_5-8_ MIF polymorphism. *Second*, the study population is relative small although it is the largest scare so far. A large and long-term follow-up study is necessary to solidate the clinical values. *Third*, we only measured plasma MIF levels in a small portion of study participants which may also limit the generalisation of our findings.

In conclusion, our findings indicate that MIF gene rs755622 variant with CC genotype and C allele is associated with increased risk of ACS. Identification of this MIF gene polymorphism may help for predicting the risk of ACS.

## Data Availability

The datasets analyzed during the current study including accession information for the raw genotyping data are available from the corresponding author on reasonable request.
